# 2,2′-Bipyridin-1′-ium 1-oxide bromide monohydrate

**DOI:** 10.1107/S2056989018002347

**Published:** 2018-02-13

**Authors:** Katharina Heintz, Helmar Görls, Wolfgang Imhof

**Affiliations:** a University Koblenz - Landau, Institute of Integrated Natural Sciences, Universitätsstrasse 1, D-56070 Koblenz, Germany; b Friedrich-Schiller-University, Insitute of Inorganic and Analytical Chemistry, Humboldtstrasse 8, D-07743 Jena, Germany

**Keywords:** crystal structure, bi­pyridine oxide, hydro­bromide, hydrate

## Abstract

The title compound 2,2′-bipyridin-1′-ium 1-oxide bromide crystallizes as a monohydrate, C_10_H_9_N_2_^+^·Br^−^·H_2_O. Structural disorder is observed due to the fact that protonation, as well as oxidation, of the N atoms of 2,2′-bi­pyridine occurs at either of the N atoms. The disorder extends to the remainder of the cation, with a refined occupancy rate of 0.717 (4) for the major moiety. An intra­molecular N—H⋯O hydrogen bond forces the bi­pyridine unit into an *s*-*cis* conformation. Each pair of neighbouring 2,2′-bipyridin-1′-ium ions forms a dimeric aggregate by hydrogen bonds between their respective N—O and the N—H functions. These dimers and hydrogen-bonding inter­actions with bromide ions and the water mol­ecule give rise to a complex supra­molecular arrangement.

## Abstract 2

Structural disorder is observed due to the fact that protonation, as well as oxidation, of the N atoms of 2,2′-bi­pyridine occurs either at either of the N atoms. The disorder extends to the remainder of the cation, with a refined occupancy rate of 0.717 (4) for the major moiety.

## Chemical context   

Bi­pyridine ligands are an important class of ligands with respect to the synthesis of transition metal complexes. They are especially well-known for their use in the development of complexes with specific photophysical (Thompson *et al.*, 2013 ▸; Sun *et al.*, 2015 ▸, Dongare *et al.*, 2017 ▸) and/or photocatalytic (Wenger, 2013 ▸; Fukuzumi *et al.*, 2016 ▸; Knoll *et al.*, 2015 ▸; Duan *et al.*, 2015 ▸; Pal & Hanan, 2014 ▸) properties or for the construction of dye-sensitized solar cells (Happ *et al.*, 2012 ▸; Bomben *et al.*, 2012 ▸; Robson *et al.*, 2012 ▸; Adeloye & Ajibade, 2014 ▸; Lu *et al.*, 2016 ▸; Omae, 2016 ▸). During our attempts to introduce substituents to 2,2′-bipyrdines that would allow us to use them as monomers in copolymerization reactions (Heintz *et al.*, 2017 ▸), we treated 2,2′-bi­pyridine with a mixture of hydro­bromic acid and hydrogen peroxide with the aim of getting direct access to 4-bromo-2,2′-bi­pyridine-1-oxide. After recrystallization, the title compound turned out to be the only isolable product.
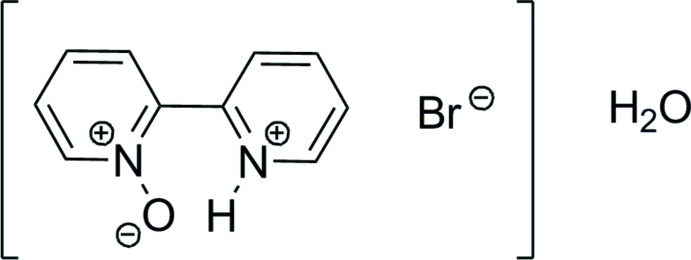


## Structural commentary   

**Figure 1:**
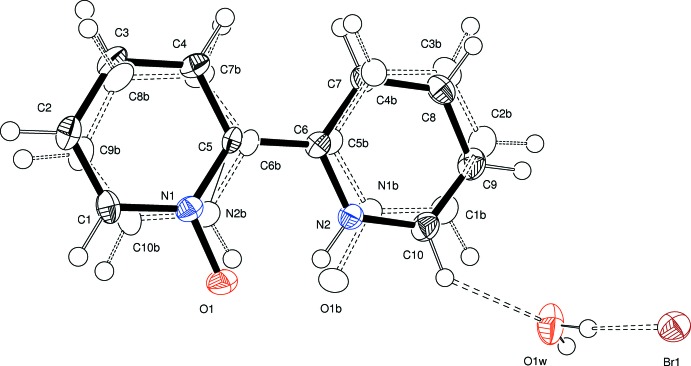
Mol­ecular structure of the cation of the title compound. Non-hydrogen atoms showing displacement ellipsoids with octand shading represent the major component of the two disordered cations.

The mol­ecular structure of the cation of the title compound is depicted in Fig. 1 ▸, showing the disorder of the cations in which the oxygen atom and the proton are bonded to either N1 or N2. The two cation moieties are disordered over the same position in an approximate 3:1 ratio, with a refined occupancy for the major moiety of 0.717 (4). The disorder has been refined in terms of a whole mol­ecule disorder, thus leading virtually identical bond lengths which, in addition, are of expected values. See the *Refinement* section for details of the refinement of the disorder. The two pyridine subunits of the 2,2′-bi­pyridine exhibit an *s*-cis conformation, which is stabilized by an intra­molecular N—H⋯O hydrogen bond (Table 1 ▸). The *s*-cis conformation also allows the cations to arrange themselves into dimeric aggregates *via* additional N—H⋯O hydrogen bonds (*cf. Supra­molecular features*).

## Supra­molecular features   

**Figure 2:**
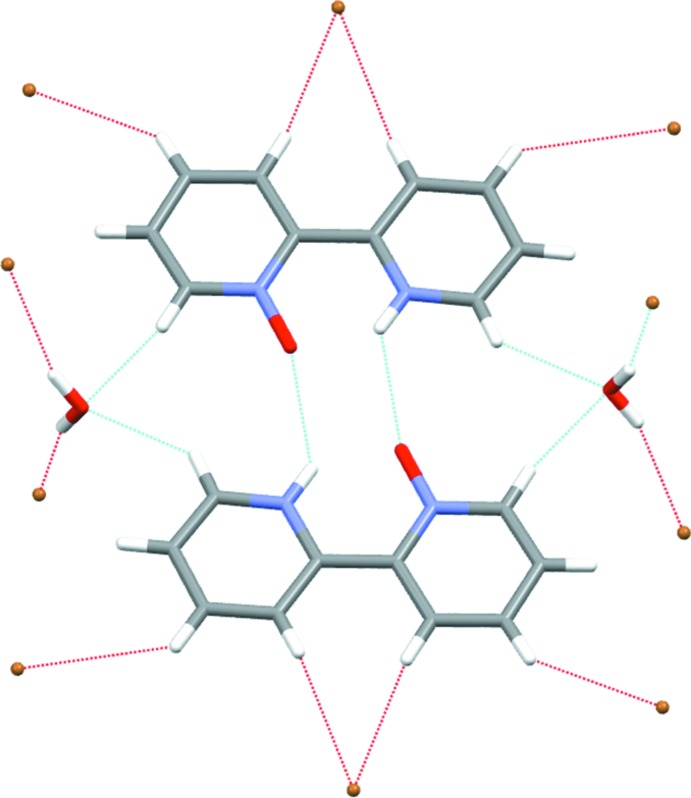
Dimer of cations formed by N—H⋯O hydrogen bonds (Table 1 ▸). Hydrogen-bonded bromide anions and water mol­ecules are also shown. Disorder of the cation is omitted for clarity.

**Figure 3:**
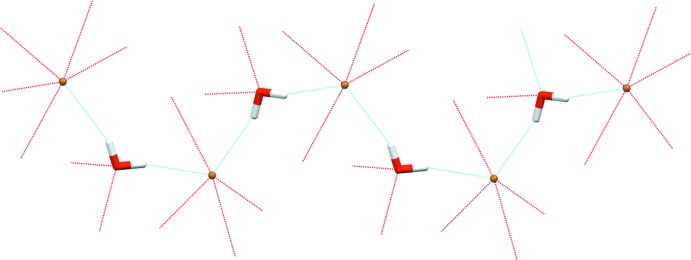
Zigzag chain of water mol­ecules and bromide anions parallel to the *b* axis.

Fig. 2 ▸ shows a dimeric aggregate built up by two cations of the title compound *via* N—H⋯O hydrogen bonds (Table 1 ▸). In addition, the figure shows that hydrogen atoms in the 3, 3′, 4 and 4′ positions of each bi­pyridine unit are engaged in C—H⋯Br hydrogen bonds (Desiraju & Steiner, 2001 ▸) with the inter­actions of the 3 and 3′ hydrogen atoms being part of a bifurcated hydrogen bond towards the bromide anion. Hydrogen atoms in the 6 and 6′ positions are part of bifurcated hydrogen bonds towards the water mol­ecule. Moreover, the hydrogen atoms of the water mol­ecules are involved in hydrogen bonds of the O—H⋯Br type. Bromide anions and water mol­ecules form zigzag chains along the *b*-axis direction (Fig. 3 ▸). In summary, a complex network structure is realized by hydrogen bonds linking the constituents of this zigzag chain into dimers of cations.

## Database survey   

According to a CSD survey (Version 5.38; Groom *et al.*, 2016 ▸) and in contrast to 2,2′-bi­pyridine or 2,2′-bi­pyridine-1-oxide, there are no metal complexes reported in which a protonated 2,2′-bi­pyridine-1-oxide acts as a ligand. Nevertheless, there are several closely related compounds that show different counter-ions. There are entries involving the hydrogensulfate (ESUMEL; Najafpour *et al.*, 2010 ▸), the perrhenate (PEPDAP; Englert *et al.*, 1993 ▸) and the triiodide (SINBIB; Lin *et al.*, 2007 ▸). All of these compounds, as well as the title compound itself, show an s-*cis* conformation of the bi­pyridine. Moreover, in all compounds, both rings of the bi­pyridine show an almost perfect coplanar arrangement with dihedral angles well below 10° [title compound: mol­ecule 1: 1.2 (6)°, mol­ecule 2: 2(2)°; ESUMEL 5.9°; PEPDAP 3.9°; SINBIB 2.7°]. This arrangement is most probably caused by the short intra­molecular N—H⋯O hydrogen bond between the protonated nitro­gen atom and the oxygen atom (title compound: mol­ecule 1 1.76, mol­ecule 2; 1.81 Å; ESUMEL 1.73 Å; PEPDAP 1.71 Å; SINBIB 1.73 Å). The supra­molecular arrangement in ESUMEL and PEPDAP is identical, with the cations also forming hydrogen-bonded dimers. Nevertheless, in contrast to the title compound, these dimers are formed by weak C—H⋯O hydrogen bonds of aromatic C—H functions towards the oxygen atom. All other hydrogen bonds are realized by oxygen atoms of the counter-ions acting as the hydrogen-bond acceptor sites. In SINBIB, the cations form an infinite plane realized by bifurcated hydrogen bonds of the oxygen atoms with aromatic C—H functions. In addition, each cation shows a weak C—H⋯I inter­action. In ESUMEL and SINBIB, the protonated N—H groups are not involved in the hydrogen-bond network, whereas in PEPDAP there is an N—H⋯O hydrogen bond to one of the perrhenate counter-ions. In summary, the hydrogen-bond network observed for the title compound is unique compared to the situation for other closely related crystal structures.

## Synthesis and crystallization   

2,2′-Bi­pyridine (1 g, 6.5 mmol) was dissolved in 15 mL methanol. Then hydro­bromic acid (0.58 g, 7.2 mmol) and a 30% solution of hydrogen peroxide (0.74 mL, 6.5 mmol) were added at 283 K. The solution was stirred at room temperature for 20 h. The clear solution turned yellow and a fine precipitate was formed, which dissolved again during the reaction time. After the solvent had evaporated, the colourless residue was dissolved in ethanol. Then water was added until a fine precipitate was formed. Storing the solution in the refrigerator (277 K) overnight led to the formation of crystals suitable for x-ray diffraction (yield: 126 mg, 0.3 mmol, 46%).

## Refinement   

Crystal data, data collection and structure refinement details are summarized in Table 2 ▸. Data were corrected for Lorentz and polarization effects. Water H atoms were freely refined All other hydrogen atoms were placed in idealized positions (N—H = 0.88, C—H = 0.95 Å) and refined using a riding model with *U*_iso_(H) = 1.2*U*_eq_(C or N). The disorder was refined in terms of a whole mol­ecule disorder. The geometry of major and minor moieties were restrained to be similar (SAME restraint in *SHELXL*) and anisotropic displacement parameters of equivalent atoms in the two moieties were constrained to be identical. Site-occupation factors of the atoms of the two disordered cations were refined using the FVAR instruction and were calculated to be 0.717 (4) (O1 to H10) and 0.283 (4) (O1*B* to H10*B*).

## Supplementary Material

Crystal structure: contains datablock(s) I. DOI: 10.1107/S2056989018002347/zl2721sup1.cif

Structure factors: contains datablock(s) I. DOI: 10.1107/S2056989018002347/zl2721Isup2.hkl

CCDC reference: 1822822

Additional supporting information:  crystallographic information; 3D view; checkCIF report

## Figures and Tables

**Table 1:** Hydrogen-bond geometry (Å, °)

*D*—H⋯*A*	*D*—H	H⋯*A*	*D*⋯*A*	*D*—H⋯*A*
N2—H1*N*2⋯O1	0.88	1.76	2.485 (4)	138
N2—H1*N*2⋯O1^i^	0.88	2.41	3.089 (5)	134
C1—H1⋯O1*W* ^i^	0.95	2.22	3.138 (6)	163
C4—H4⋯Br1^ii^	0.95	2.75	3.687 (10)	167
C7—H7⋯Br1^ii^	0.95	2.86	3.769 (10)	160
C10—H10⋯O1*W*	0.95	2.34	3.074 (5)	134
N2*B*—H2*N*2⋯O1*B*	0.88	1.81	2.500 (15)	134
N2*B*—H2*N*2⋯O1*B* ^i^	0.88	2.35	3.117 (18)	146
C1*B*—H1*B*⋯O1*W*	0.95	2.09	2.979 (14)	156
C2*B*—H2*B*⋯Br1^iii^	0.95	2.80	3.497 (15)	131
C4*B*—H4*B*⋯Br1^ii^	0.95	2.77	3.70 (3)	168
C7*B*—H7*B*⋯Br1^ii^	0.95	2.92	3.80 (3)	155
C10*B*—H10*B*⋯O1*W* ^i^	0.95	2.40	3.246 (17)	147
O1*W*—H1*W*1⋯Br1	0.90 (3)	2.47 (3)	3.3475 (18)	165 (3)
O1*W*—H2*W*1⋯Br1^iv^	0.84 (3)	2.57 (3)	3.3754 (17)	160 (3)

Symmetry codes: (i) 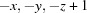; (ii) 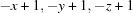; (iii) 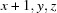; (iv) 
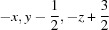
.

**Table 2:** Experimental details

Crystal data	
Chemical formula	C_10_H_9_N_2_O^+^·Br^−^·H_2_O
*M*_r_	271.12
Crystal system, space group	Monoclinic, *P*2_1_/*c*
Temperature (K)	133
*a*, *b*, *c* (Å)	5.7882 (1), 9.2095 (2), 20.2485 (4)
β (°)	91.701 (1)
*V* (Å^3^)	1078.90 (4)
*Z*	4
Radiation type	Mo *K*α
μ (mm^−1^)	3.79
Crystal size (mm)	0.05 × 0.04 × 0.03

Data collection	
Diffractometer	Nonius KappaCCD
Absorption correction	Multi-scan (*SADABS*; Sheldrick, 2002 ▸)
*T*_min_, *T*_max_	0.557, 0.746
No. of measured, independent and observed [*I* > 2σ(*I*)] reflections	12419, 2465, 2226
*R*_int_	0.030
(sin θ/λ)_max_ (Å^−1^)	0.649

Refinement	
*R*[*F* ^2^ > 2σ(*F* ^2^)], *wR*(*F* ^2^), *S*	0.023, 0.052, 1.07
No. of reflections	2465
No. of parameters	190
No. of restraints	32
H-atom treatment	H atoms treated by a mixture of independent and constrained refinement
Δρ_max_, Δρ_min_ (e Å^−3^)	0.46, −0.51

Computer programs: *COLLECT* (Nonius, 1998 ▸), *DENZO* (Otwinowski & Minor, 1997 ▸), *SHELXS97* (Sheldrick, 2008), *SHELXL2018* (Sheldrick, 2015 ▸), *ORTEP-3 for Windows* (Farrugia, 2012 ▸) and *Mercury* (Macrae *et al.*, 2006 ▸).

## supplementary crystallographic information

### Crystal data

**Table:**

C_10_H_9_N_2_O^+^·Br^−^·H_2_O	*F*(000) = 544
*M**_r_* = 271.12	*D*_x_ = 1.669 Mg m^−^^3^
Monoclinic, *P*2_1_/*c*	Mo *K*α radiation, λ = 0.71073 Å
*a* = 5.7882 (1) Å	Cell parameters from 12419 reflections
*b* = 9.2095 (2) Å	θ = 2.0–27.5°
*c* = 20.2485 (4) Å	µ = 3.79 mm^−^^1^
β = 91.701 (1)°	*T* = 133 K
*V* = 1078.90 (4) Å^3^	Prism, colourless
*Z* = 4	0.05 × 0.04 × 0.03 mm

### Data collection

**Table:**

Nonius KappaCCD diffractometer	2465 independent reflections
Radiation source: fine-focus sealed tube	2226 reflections with *I* > 2σ(*I*)
Graphite monochromator	*R*_int_ = 0.030
phi– + ω–scan	θ_max_ = 27.5°, θ_min_ = 2.0°
Absorption correction: multi-scan (SADABS; Sheldrick, 2002)	*h* = −7→7
*T*_min_ = 0.557, *T*_max_ = 0.746	*k* = −9→11
12419 measured reflections	*l* = −26→26

### Refinement

**Table:**

Refinement on *F*^2^	Primary atom site location: structure-invariant direct methods
Least-squares matrix: full	Secondary atom site location: difference Fourier map
*R*[*F*^2^ > 2σ(*F*^2^)] = 0.023	Hydrogen site location: mixed
*wR*(*F*^2^) = 0.052	H atoms treated by a mixture of independent and constrained refinement
*S* = 1.07	*w* = 1/[σ^2^(*F*_o_^2^) + (0.0113*P*)^2^ + 0.9881*P*] where *P* = (*F*_o_^2^ + 2*F*_c_^2^)/3
2465 reflections	(Δ/σ)_max_ = 0.001
190 parameters	Δρ_max_ = 0.46 e Å^−^^3^
32 restraints	Δρ_min_ = −0.51 e Å^−^^3^

### Special details

**Table:**

Geometry. All esds (except the esd in the dihedral angle between two l.s. planes) are estimated using the full covariance matrix. The cell esds are taken into account individually in the estimation of esds in distances, angles and torsion angles; correlations between esds in cell parameters are only used when they are defined by crystal symmetry. An approximate (isotropic) treatment of cell esds is used for estimating esds involving l.s. planes.
Refinement. Refinement of F^2^ against ALL reflections. The weighted R-factor wR and goodness of fit S are based on F^2^, conventional R-factors R are based on F, with F set to zero for negative F^2^. The threshold expression of F^2^ > 2sigma(F^2^) is used only for calculating R-factors(gt) etc. and is not relevant to the choice of reflections for refinement. R-factors based on F^2^ are statistically about twice as large as those based on F, and R- factors based on ALL data will be even larger.

### Fractional atomic coordinates and isotropic or equivalent isotropic displacement parameters (Å^2^)

**Table:**

	*x*	*y*	*z*	*U*_iso_*/*U*_eq_	Occ. (<1)
Br1	−0.09302 (3)	0.63110 (2)	0.65415 (2)	0.02577 (7)	
O1	0.0960 (3)	−0.0036 (2)	0.43461 (9)	0.0270 (5)	0.717 (4)
N1	0.2649 (12)	0.0073 (8)	0.3908 (2)	0.0199 (9)	0.717 (4)
N2	0.3050 (9)	0.1763 (6)	0.5057 (2)	0.0206 (7)	0.717 (4)
H1N2	0.188837	0.116543	0.497651	0.025*	0.717 (4)
C1	0.2378 (9)	−0.0729 (6)	0.33445 (16)	0.0236 (8)	0.717 (4)
H1	0.103588	−0.130999	0.327701	0.028*	0.717 (4)
C2	0.4039 (8)	−0.0697 (6)	0.2875 (2)	0.0252 (9)	0.717 (4)
H2	0.385065	−0.126508	0.248510	0.030*	0.717 (4)
C3	0.5987 (11)	0.0160 (10)	0.2967 (4)	0.0254 (8)	0.717 (4)
H3	0.716054	0.016114	0.264870	0.031*	0.717 (4)
C4	0.620 (2)	0.1013 (15)	0.3526 (4)	0.0211 (11)	0.717 (4)
H4	0.748457	0.165180	0.358187	0.025*	0.717 (4)
C5	0.4539 (15)	0.0933 (9)	0.4009 (4)	0.0162 (9)	0.717 (4)
C6	0.4718 (14)	0.1856 (12)	0.4606 (3)	0.0174 (9)	0.717 (4)
C7	0.6635 (19)	0.2733 (8)	0.4752 (5)	0.0217 (11)	0.717 (4)
H7	0.791700	0.274957	0.447024	0.026*	0.717 (4)
C8	0.6624 (8)	0.3582 (9)	0.5321 (3)	0.0254 (9)	0.717 (4)
H8	0.783428	0.426128	0.540232	0.031*	0.717 (4)
C9	0.4864 (8)	0.3452 (6)	0.5776 (3)	0.0276 (11)	0.717 (4)
H9	0.491253	0.398271	0.617799	0.033*	0.717 (4)
C10	0.3066 (7)	0.2532 (5)	0.5621 (2)	0.0254 (9)	0.717 (4)
H10	0.182364	0.243774	0.591417	0.030*	0.717 (4)
O1B	0.1468 (8)	0.1100 (5)	0.5162 (2)	0.0283 (14)	0.283 (4)
N1B	0.330 (3)	0.1968 (18)	0.5193 (7)	0.0206 (7)	0.283 (4)
N2B	0.263 (3)	0.018 (2)	0.4059 (7)	0.0199 (9)	0.283 (4)
H2N2	0.165293	0.014377	0.438408	0.024*	0.283 (4)
C1B	0.361 (2)	0.2832 (16)	0.5724 (6)	0.0254 (9)	0.283 (4)
H1B	0.251328	0.281476	0.606250	0.030*	0.283 (4)
C2B	0.546 (3)	0.3722 (19)	0.5783 (8)	0.0276 (11)	0.283 (4)
H2B	0.556963	0.436182	0.615077	0.033*	0.283 (4)
C3B	0.716 (3)	0.373 (3)	0.5334 (10)	0.0254 (9)	0.283 (4)
H3B	0.858710	0.421752	0.541526	0.031*	0.283 (4)
C4B	0.668 (5)	0.299 (3)	0.4753 (13)	0.0217 (11)	0.283 (4)
H4B	0.756541	0.319587	0.437556	0.026*	0.283 (4)
C5B	0.493 (4)	0.196 (3)	0.4710 (10)	0.0174 (9)	0.283 (4)
C6B	0.450 (4)	0.112 (3)	0.4118 (11)	0.0162 (9)	0.283 (4)
C7B	0.607 (6)	0.094 (4)	0.3618 (13)	0.0211 (11)	0.283 (4)
H7B	0.754015	0.139492	0.366763	0.025*	0.283 (4)
C8B	0.558 (3)	0.015 (3)	0.3049 (11)	0.0254 (8)	0.283 (4)
H8B	0.657616	0.018628	0.268447	0.031*	0.283 (4)
C9B	0.361 (3)	−0.0716 (19)	0.3024 (7)	0.0252 (9)	0.283 (4)
H9B	0.324956	−0.132271	0.265541	0.030*	0.283 (4)
C10B	0.224 (3)	−0.0649 (19)	0.3544 (5)	0.0236 (8)	0.283 (4)
H10B	0.089399	−0.124292	0.353836	0.028*	0.283 (4)
O1W	0.1320 (3)	0.31147 (18)	0.70149 (8)	0.0383 (4)	
H1W1	0.067 (6)	0.400 (4)	0.6970 (16)	0.075 (10)*	
H2W1	0.117 (5)	0.288 (3)	0.7415 (16)	0.064 (9)*	

### Atomic displacement parameters (Å^2^)

**Table:**

	*U*^11^	*U*^22^	*U*^33^	*U*^12^	*U*^13^	*U*^23^
Br1	0.02760 (10)	0.02744 (12)	0.02241 (10)	0.00035 (7)	0.00334 (7)	0.00392 (7)
O1	0.0252 (10)	0.0336 (11)	0.0225 (10)	−0.0067 (8)	0.0068 (7)	−0.0001 (8)
N1	0.0208 (8)	0.0207 (15)	0.019 (3)	0.0010 (8)	0.0034 (18)	0.002 (2)
N2	0.0220 (15)	0.024 (2)	0.016 (2)	−0.0026 (11)	0.0018 (14)	−0.0009 (14)
C1	0.0280 (13)	0.0231 (12)	0.019 (2)	−0.0009 (10)	−0.006 (2)	−0.004 (2)
C2	0.030 (2)	0.0272 (11)	0.018 (2)	0.0045 (15)	0.0008 (13)	−0.0047 (17)
C3	0.022 (3)	0.0337 (11)	0.021 (2)	0.0066 (19)	0.0064 (15)	−0.0024 (15)
C4	0.0189 (17)	0.0273 (17)	0.017 (3)	0.0011 (11)	0.0040 (17)	0.003 (2)
C5	0.0186 (8)	0.015 (3)	0.015 (3)	0.0039 (14)	0.0018 (16)	−0.0031 (14)
C6	0.0199 (18)	0.0198 (19)	0.012 (3)	0.0021 (12)	−0.0014 (18)	0.0032 (18)
C7	0.0259 (10)	0.017 (3)	0.0225 (9)	0.000 (2)	0.0028 (7)	0.0005 (19)
C8	0.025 (3)	0.025 (2)	0.0259 (10)	−0.004 (2)	−0.005 (2)	−0.0014 (11)
C9	0.037 (3)	0.028 (3)	0.0183 (10)	0.000 (2)	0.0029 (19)	−0.0003 (15)
C10	0.030 (2)	0.029 (2)	0.0179 (18)	−0.0004 (15)	0.0043 (14)	−0.0005 (14)
O1B	0.025 (2)	0.034 (3)	0.026 (3)	−0.011 (2)	0.0061 (19)	−0.004 (2)
N1B	0.0220 (15)	0.024 (2)	0.016 (2)	−0.0026 (11)	0.0018 (14)	−0.0009 (14)
N2B	0.0208 (8)	0.0207 (15)	0.019 (3)	0.0010 (8)	0.0034 (18)	0.002 (2)
C1B	0.030 (2)	0.029 (2)	0.0179 (18)	−0.0004 (15)	0.0043 (14)	−0.0005 (14)
C2B	0.037 (3)	0.028 (3)	0.0183 (10)	0.000 (2)	0.0029 (19)	−0.0003 (15)
C3B	0.025 (3)	0.025 (2)	0.0259 (10)	−0.004 (2)	−0.005 (2)	−0.0014 (11)
C4B	0.0259 (10)	0.017 (3)	0.0225 (9)	0.000 (2)	0.0028 (7)	0.0005 (19)
C5B	0.0199 (18)	0.0198 (19)	0.012 (3)	0.0021 (12)	−0.0014 (18)	0.0032 (18)
C6B	0.0186 (8)	0.015 (3)	0.015 (3)	0.0039 (14)	0.0018 (16)	−0.0031 (14)
C7B	0.0189 (17)	0.0273 (17)	0.017 (3)	0.0011 (11)	0.0040 (17)	0.003 (2)
C8B	0.022 (3)	0.0337 (11)	0.021 (2)	0.0066 (19)	0.0064 (15)	−0.0024 (15)
C9B	0.030 (2)	0.0272 (11)	0.018 (2)	0.0045 (15)	0.0008 (13)	−0.0047 (17)
C10B	0.0280 (13)	0.0231 (12)	0.019 (2)	−0.0009 (10)	−0.006 (2)	−0.004 (2)
O1W	0.0614 (10)	0.0280 (8)	0.0259 (8)	0.0023 (8)	0.0041 (7)	−0.0044 (7)

### Geometric parameters (Å, º)

**Table:**

O1—N1	1.343 (6)	N1B—C1B	1.345 (12)
N1—C5	1.361 (7)	N1B—C5B	1.378 (19)
N1—C1	1.364 (4)	N2B—C10B	1.307 (12)
N2—C10	1.344 (4)	N2B—C6B	1.386 (19)
N2—C6	1.351 (7)	N2B—H2N2	0.8800
N2—H1N2	0.8800	C1B—C2B	1.347 (11)
C1—C2	1.373 (4)	C1B—H1B	0.9500
C1—H1	0.9500	C2B—C3B	1.362 (17)
C2—C3	1.384 (7)	C2B—H2B	0.9500
C2—H2	0.9500	C3B—C4B	1.38 (2)
C3—C4	1.381 (8)	C3B—H3B	0.9500
C3—H3	0.9500	C4B—C5B	1.392 (19)
C4—C5	1.396 (7)	C4B—H4B	0.9500
C4—H4	0.9500	C5B—C6B	1.444 (12)
C5—C6	1.478 (4)	C6B—C7B	1.390 (19)
C6—C7	1.397 (8)	C7B—C8B	1.39 (2)
C7—C8	1.393 (8)	C7B—H7B	0.9500
C7—H7	0.9500	C8B—C9B	1.390 (17)
C8—C9	1.398 (6)	C8B—H8B	0.9500
C8—H8	0.9500	C9B—C10B	1.339 (11)
C9—C10	1.371 (4)	C9B—H9B	0.9500
C9—H9	0.9500	C10B—H10B	0.9500
C10—H10	0.9500	O1W—H1W1	0.90 (3)
O1B—N1B	1.330 (12)	O1W—H2W1	0.84 (3)
			
O1—N1—C5	122.8 (4)	O1B—N1B—C5B	121.8 (11)
O1—N1—C1	116.4 (5)	C1B—N1B—C5B	119.5 (12)
C5—N1—C1	120.8 (5)	C10B—N2B—C6B	123.3 (15)
C10—N2—C6	123.6 (4)	C10B—N2B—H2N2	118.4
C10—N2—H1N2	118.2	C6B—N2B—H2N2	118.4
C6—N2—H1N2	118.2	N1B—C1B—C2B	121.2 (14)
N1—C1—C2	120.2 (5)	N1B—C1B—H1B	119.4
N1—C1—H1	119.9	C2B—C1B—H1B	119.4
C2—C1—H1	119.9	C1B—C2B—C3B	122.1 (15)
C1—C2—C3	120.2 (4)	C1B—C2B—H2B	119.0
C1—C2—H2	119.9	C3B—C2B—H2B	119.0
C3—C2—H2	119.9	C2B—C3B—C4B	115.9 (15)
C4—C3—C2	119.3 (5)	C2B—C3B—H3B	122.1
C4—C3—H3	120.4	C4B—C3B—H3B	122.1
C2—C3—H3	120.4	C3B—C4B—C5B	121 (2)
C3—C4—C5	119.8 (7)	C3B—C4B—H4B	119.4
C3—C4—H4	120.1	C5B—C4B—H4B	119.4
C5—C4—H4	120.1	N1B—C5B—C4B	117.6 (17)
N1—C5—C4	119.6 (5)	N1B—C5B—C6B	119.0 (17)
N1—C5—C6	119.6 (6)	C4B—C5B—C6B	121.9 (19)
C4—C5—C6	120.6 (7)	N2B—C6B—C7B	113.1 (16)
N2—C6—C7	118.2 (6)	N2B—C6B—C5B	121.5 (18)
N2—C6—C5	118.8 (6)	C7B—C6B—C5B	124 (2)
C7—C6—C5	122.8 (7)	C8B—C7B—C6B	123 (2)
C8—C7—C6	118.5 (8)	C8B—C7B—H7B	118.5
C8—C7—H7	120.7	C6B—C7B—H7B	118.5
C6—C7—H7	120.7	C7B—C8B—C9B	118.6 (16)
C7—C8—C9	121.2 (5)	C7B—C8B—H8B	120.7
C7—C8—H8	119.4	C9B—C8B—H8B	120.7
C9—C8—H8	119.4	C10B—C9B—C8B	116.8 (14)
C10—C9—C8	117.8 (5)	C10B—C9B—H9B	121.6
C10—C9—H9	121.1	C8B—C9B—H9B	121.6
C8—C9—H9	121.1	N2B—C10B—C9B	124.2 (16)
N2—C10—C9	120.4 (4)	N2B—C10B—H10B	117.9
N2—C10—H10	119.8	C9B—C10B—H10B	117.9
C9—C10—H10	119.8	H1W1—O1W—H2W1	106 (3)
O1B—N1B—C1B	118.7 (12)		

### Hydrogen-bond geometry (Å, º)

**Table:**

*D*—H···*A*	*D*—H	H···*A*	*D*···*A*	*D*—H···*A*
N2—H1*N*2···O1	0.88	1.76	2.485 (4)	138
N2—H1*N*2···O1^i^	0.88	2.41	3.089 (5)	134
C1—H1···O1*W*^i^	0.95	2.22	3.138 (6)	163
C4—H4···Br1^ii^	0.95	2.75	3.687 (10)	167
C7—H7···Br1^ii^	0.95	2.86	3.769 (10)	160
C10—H10···O1*W*	0.95	2.34	3.074 (5)	134
N2*B*—H2*N*2···O1*B*	0.88	1.81	2.500 (15)	134
N2*B*—H2*N*2···O1*B*^i^	0.88	2.35	3.117 (18)	146
C1*B*—H1*B*···O1*W*	0.95	2.09	2.979 (14)	156
C2*B*—H2*B*···Br1^iii^	0.95	2.80	3.497 (15)	131
C4*B*—H4*B*···Br1^ii^	0.95	2.77	3.70 (3)	168
C7*B*—H7*B*···Br1^ii^	0.95	2.92	3.80 (3)	155
C10*B*—H10*B*···O1*W*^i^	0.95	2.40	3.246 (17)	147
O1*W*—H1*W*1···Br1	0.90 (3)	2.47 (3)	3.3475 (18)	165 (3)
O1*W*—H2*W*1···Br1^iv^	0.84 (3)	2.57 (3)	3.3754 (17)	160 (3)

Symmetry codes: (i) −*x*, −*y*, −*z*+1; (ii) −*x*+1, −*y*+1, −*z*+1; (iii) *x*+1, *y*, *z*; (iv) −*x*, *y*−1/2, −*z*+3/2.
